# Combination of High-density Microelectrode Array and Patch Clamp Recordings to Enable Studies of Multisynaptic Integration

**DOI:** 10.1038/s41598-017-00981-4

**Published:** 2017-04-20

**Authors:** David Jäckel, Douglas J. Bakkum, Thomas L. Russell, Jan Müller, Milos Radivojevic, Urs Frey, Felix Franke, Andreas Hierlemann

**Affiliations:** ETH Zurich, Department of Biosystems Science and Engineering, 4058 Basel, Switzerland

## Abstract

We present a novel, all-electric approach to record and to precisely control the activity of tens of individual presynaptic neurons. The method allows for parallel mapping of the efficacy of multiple synapses and of the resulting dynamics of postsynaptic neurons in a cortical culture. For the measurements, we combine an extracellular high-density microelectrode array, featuring 11’000 electrodes for extracellular recording and stimulation, with intracellular patch-clamp recording. We are able to identify the contributions of individual presynaptic neurons - including inhibitory and excitatory synaptic inputs - to postsynaptic potentials, which enables us to study dendritic integration. Since the electrical stimuli can be controlled at microsecond resolution, our method enables to evoke action potentials at tens of presynaptic cells in precisely orchestrated sequences of high reliability and minimum jitter. We demonstrate the potential of this method by evoking short- and long-term synaptic plasticity through manipulation of multiple synaptic inputs to a specific neuron.

## Introduction

Simultaneous recordings of pre- and postsynaptic neurons are crucial for studying the fundamental properties of neural signal transmission. In paired-recordings, for example, a presynaptic and a postsynaptic neuron are simultaneously patch-clamped, and action potentials (APs) are evoked in the presynaptic cell that then triggers signals in the postsynaptic cell. Paired-recordings were used to study, e.g., short-term plasticity of inhibitory^1^ and excitatory^2^ synaptic connections as well as for investigating long-term plasticity effects, such as long-term potentiation (LTP), or long-term depression (LTD)^3^. By stimulating both, pre- and postsynaptic cell, with controlled temporal delay, spike-timing dependent plasticity (STDP) can be evoked^4, 5^.

Cortical neurons, however, typically receive synaptic input from many, up to thousands of cells. For this reason, a key question to understanding neural computation is how *multiple* synaptic inputs integrate and interact within the postsynaptic cell. Many interacting effects between individual synaptic inputs have been identified, such as nonlinear summation properties of multiple inputs through dendrites^6–8^, temporal precision and reliability of combined synaptic inputs^9^, heterosynaptic long-term^10, 11^ and short-term plasticity effects^12^.

Nevertheless, only few electrophysiological techniques have been introduced that allow for measuring synaptic inputs from multiple presynaptic cells. Paired-recordings from multiple, simultaneously patch-clamped neurons represent a powerful measurement configuration, as multidirectional connectivity can be accurately characterized. Some studies have reported as many as 8–12 neurons that were simultaneously recorded in brain slices^13, 14^. Reliably achieving such large numbers of simultaneously patch-clamped neurons is, however, extremely challenging and requires highly specialized recording setups and technical skills. Only a few laboratories have established recordings from more than 3–4 simultaneously patched cells.

An alternative approach includes to patch-clamp an individual neuron while, at the same time, APs of presynaptic cells are detected or actively evoked. The presynaptic spike times and stimulation timings are then used for spike-triggered-averaging (STA) of the intracellular signal of the patched cell in order to compute the average postsynaptic potentials (PSPs). The ‘reverse optical probing‘ technique^15, 16^ applies calcium imaging and reverse correlation analysis to identify neurons that fire APs time-locked with detected synaptic events. Another approach is ‘photostimulation scanning’^17^, where photolytic release of caged glutamate is applied to sequentially stimulate neurons while an individual neuron is recorded from intracellularly. The stimulation of presynaptic cells can also be carried out by electrical means with individual extracellular bipolar electrodes^11, 12^, or with arrays of stimulating microelectrodes^10^. Optical approaches typically suffer from limited temporal resolution, from difficulty to accurately evoke and record single presynaptic APs and from chemical side-effects or phototoxicity. Electrical stimulation techniques, on the other hand, have been substantially limited with regard to the number of activated neurons, which depends on number and dimension of available stimulation electrodes.

Recently developed planar high-density microelectrode arrays (HD-MEAs), based on complementary metal oxide semiconductor (CMOS) technology^18–23^ feature several tens of thousands of electrodes at cellular and subcellular spatial resolution. In this work, we show that HD-MEA recordings can be combined with the patch clamp technique, in order to precisely map and stimulate synaptic connections in networks of cultured cortical neurons. The HD-MEA used here^22^ featured 11,011 densely packed electrodes in an area of 1.99 × 1.75 mm^2^, which could be used for electrical stimulation and recording of neuronal activity (up to 126 recording electrodes simultaneously). Due to the closely spaced microelectrodes, activity of individual neurons was always recorded by multiple electrodes, which greatly improved spike sorting performance^24–27^. We present two methods to study connectivity and multi-synaptic integration within a neuronal culture.

In the first approach, the possibility to map synaptic signals based on spontaneous extracellular activity measurements was explored. APs of many neurons were recorded with the HD-MEA, while, at the same time, individual neurons were patch-clamped. We then spike-sorted the extracellular data and searched for temporal correlations between the synaptic activity of the patched postsynaptic neuron and the firing patterns of extracellularly recorded neurons. If a constant delay and low jitter was found between extracellular AP and PSP, it was identified as a direct synaptic connection between the pre- and postsynaptic neuron.

Every electrode of the HD-MEA can also be used to apply precisely timed and spatially confined stimuli^28, 29^. The second approach exploits this stimulation capability: While performing intracellular recording on a selected neuron, we used extracellular stimulation to find electrodes that stimulated specific neurons, which, in turn, elicited a PSP in the patched cell. A semi-automatic algorithm was then used to identify the electrodes that reliably evoked monosynaptic PSPs in the patched neuron. We employed this method to demonstrate simultaneous short- and long-term plasticity measurements from eight synaptic inputs to a specific neuron.

## Results

**Figure 1:**
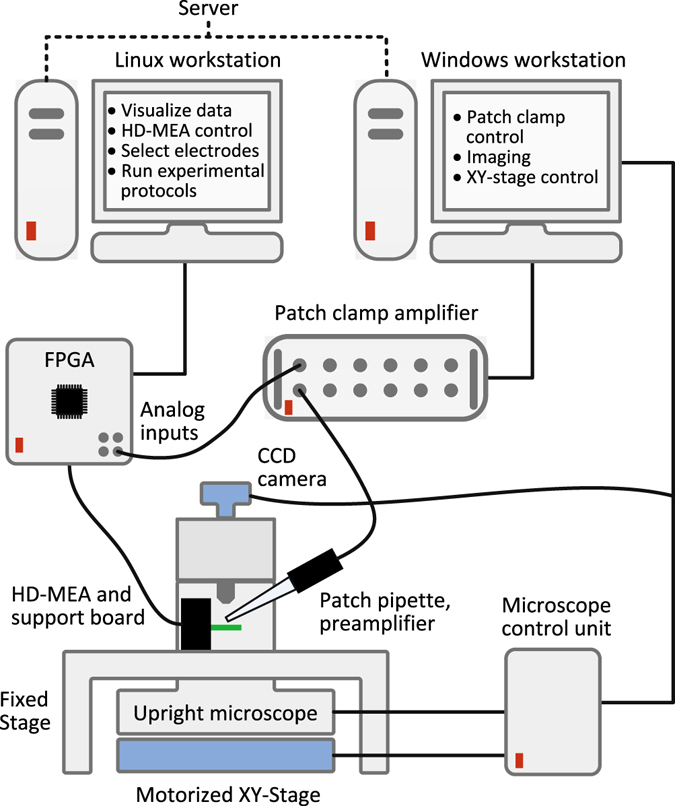
Experimental setup. Experimental setup consisting of a HD-MEA system, a patch clamp system, and an upright microscope. The HD-MEA is controlled through an FPGA and a Linux computer. A windows computer controls the patch-clamp amplifier settings and the microscope. The microscope is mounted on an XY-stage, which allows for determining the exact positioning for every acquired image. The Linux workstation is used to record the combined intra- and extracellular data, to visualize the data, to align acquired images to the MEA coordinates, and to run the experimental protocols.

We sought to combine the advantages of intracellular and extracellular HD-MEA electrophysiology by building a set-up that allowed us to patch cultured neurons directly on top of HD-MEAs under visual supervision. Figure 1 shows a diagram of the measurement set-up with the individual components.

**Figure 2:**
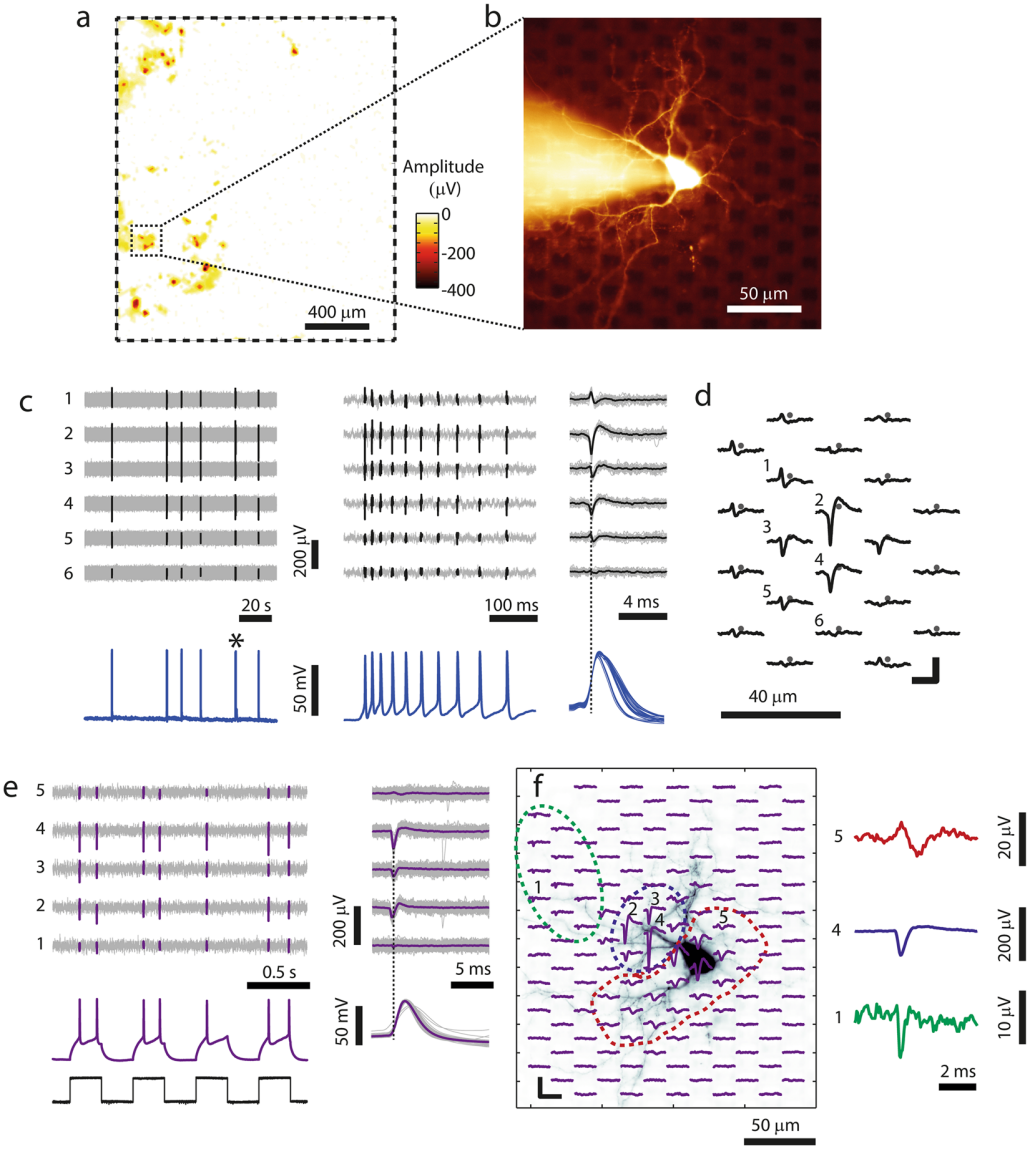
Patch-clamping of neurons on top of HD-MEAs. (**a**) Spontaneous spike amplitude map, with the color code indicating the negative-peak signal at each electrode. The electrode array outline is indicated by the dashed rectangle. (**b**) Fluorescence image of a neuron on the array, patched in the whole-cell configuration. In the background, the Pt-black-covered electrodes can be seen as black squares. (**c**) *Left*: Intracellular (*bottom*) and extracellular recordings from six selected MEA electrodes (*top*) of another example neuron. During the two minutes of displayed electrical activity, the neuron spontaneously fired APs in six bursts. *Center*: Close-up view of one individual burst marked with an asterisk in the left panel. *Right*: superposition of all detected waveforms where the black traces represent the spike-triggered average (STA) waveforms. The dashed vertical lines in (**c**) and (**e**) were aligned with the negative peak of the largest extracellular spike for timing visualization. (**d**) Footprint (spatially distributed STA signals) of the neuron in (**c**) with the grey dots representing electrode positions. The numbers mark the 6 electrodes that produced the signals displayed in (**c**). Scale bars: 2 ms/50 µV. (**e**) Extracellular and intracellular recordings from the neuron displayed in (**f**), the recording electrodes are labeled 1–5 in (**f**). *Left*: APs were evoked by injecting current pulses of 100 pA (250-ms pulses at 2 Hz, black signal at the *bottom*), where every pulse evoked 1–2 APs. *Right*: Extracellular and intracellular STA waveforms of the evoked spikes. (**f**) *Left:* Footprint superimposed to a fluorescence image of the patched neuron, scale bars: 100 µV/5 ms. *Right:* Magnified spike signals representing typical axonal (*electrode 1*, green), somatic (*electrode 4*, blue) and dendritic (*electrode 5*, red) shapes. Note that the neuron in (**e**,**f**) is the same as displayed in (**b**).

Typically after two weeks *in vitro*, the networks of dissociated cortical neurons featured regular spontaneous spiking activity in the incubator. Figure 2a shows a spontaneous spike amplitude map (SSAM) of neuronal activity recorded by the HD-MEA, represented by the largest negative-peak signal for every electrode. The SSAMs were acquired by scanning the entire chip area with high-density electrode blocks, typically prior to the patch-clamp experiments while the neuronal culture remained in the incubator. Since the SSAM was acquired noninvasively and without the need of optical means, we were able to continuously assess the overall viability and activity of the culture during the growth phase, as well as to identify promising areas of interest for the patch-clamp experiments.

The next step was to use the information from the SSAM to target specific areas on the HD-MEA for patching neurons. To this end, an automated coordinate mapping method was incorporated into the system, which allowed us to automatically drive the microscope to any selected position within the electrode array area. By using this feature, promising areas in the SSAM were identified, localized, and we then searched for cells by means of microscopy. We successfully patched neurons on HD-MEAs by visualizing them with an upright microscope equipped with difference interference contrast (DIC) optics, which yielded sufficient visibility to perform visually-guided patch clamp recordings (Supplementary Figure 1). Figure 2b shows the fluorescence image of a neuron, which was targeted for patch-clamping based on the SSAM in Fig. 2a. The Pt-black-covered electrodes of the MEA can be seen as dark squares in the background.

Once a neuron had been patched, the electrodes in its close vicinity were automatically identified, and the HD-MEA was reprogrammed so that these neighboring electrodes were used for extracellular recording. For a spontaneously firing neuron, Fig. 2c (*left and center panels*) shows the simultaneously recorded intracellular (*bottom*) and extracellular (*top*) signals. The intracellular APs allowed us to detect spike times without the need of applying spike sorting techniques. We then used the spike timings to compute the spike-triggered average (STA) extracellular signal, which is the mean extracellular spike, for every recording electrode (Fig. 2c *right*). The extracellular signal of patched cells was always visible on multiple electrodes, as shown in the examples in Fig. 2c,d and e. In Fig. 2d, the STA extracellular signals of the individual electrodes were spatially arranged according to the respective electrode positions. This spatial distribution of STA signals represents a cell-specific, extracellular neuronal signature and will be referred to as *footprint* ^28, 30^.

Many times, the patched neurons did not fire spontaneously, fired at very low rates, or fired only during network bursts. In order to obtain the footprint in these cases, we actively evoked APs by intracellular current injection. An example of such a neuron is visualized in Fig. 2e,f, where current pulses were applied with the current adjusted to evoke 1–2 APs per pulse (Fig. 2e *left bottom*). At the same time, the area surrounding the patched cell was scanned for extracellular activity. The STA traces and the footprint, which was computed based on the AP spike times in the intracellular signal, are shown in Fig. 2e,f. This method allowed us to obtain the intracellular signal, a mapping of the extracellular footprint, and the neuronal morphology for every patched cell on the array. As visualized in Fig. 2f, the footprint represents an “electrical image” of the neuron at high spatiotemporal resolution and includes signals from different subcellular compartments, which can be distinguished based on their spike shapes^31^. On the right side of Fig. 2f, three examples of spike signals are magnified: A low-magnitude fast axonal spike (green), a somatic spike with large negative-first deflection) and a dendritic spike (broader signal with positive-first deflection). The dashed areas in Fig. 2f mark the axonal (*green*), somatic (*blue*) and dendritic (*red*) compartments in the electrical footprint and enable comparison with the optical image.

The synchronization of the intra- and the extracellular signal was ensured by integrating the patch-clamp and the HD-MEA signals and recording them through the same data acquisition system, as illustrated in Fig. 1. Besides temporal synchronization, this feature allowed us to compare extra- and intracellular APs in real-time during the experiments and to integrate signal acquisition and data analysis tools into one overarching software environment.

In the following, we will analyze the results of the two methods for mapping synaptic connections based on combining the HD-MEA with the patch clamp technique. First, in *Method 1* we exploited the capability to simultaneously record extracellular signals from populations of distributed neurons by means of the HD-MEA, and to record, at the same time, the intracellular signal of an individual patched cell. Spike sorting and spike-triggered-averaging techniques enabled us to identify neuronal units with direct synaptic connections to the patched cell and to measure the postsynaptic potentials (PSPs).

The second approach, *Method 2*, relied on the stimulation capabilities of the HD-MEA. APs in multiple neurons were evoked by sequential voltage stimulation across hundreds of HD-MEA electrodes with different stimulation amplitudes, while a single neuron was patched. We then used stimulation-triggered averaging to identify which electrodes evoked monosynaptic PSPs through the respective presynaptic neurons.

### Method 1: Mapping PSPs based on Spontaneous Presynaptic Spikes

The HD-MEA system provides the possibility to actively select subsets of electrodes with great flexibility. Therefore, besides recording individual neurons by using multiple electrodes, as shown in Fig. 2, the chip can also be used to simultaneously record from multiple neurons in the network. We used this capability to record the spiking activity of multiple neurons while the intracellular signal of one particular patched cell was measured.

First, while remaining in the incubator, the array was scanned for spontaneous activity, and the SSAM was computed. We then detected local peaks of large spike amplitudes in the SSAM (see Methods). The electrodes at amplitude peaks represented attractive recording sites, as large extracellular spikes could be detected. In the next step, we re-programmed the array routing configuration to simultaneously record from 2–4 neighbored electrodes at every identified site of large activity. Multiple electrodes per location were chosen, since the spike sorting performance could be significantly increased by measuring extracellular signals through multiple, closely-spaced electrodes as compared to just using individual electrodes^24–27^. The chip was afterwards transferred to the patch-clamp measurement set-up, where individual neurons were patched.

**Figure 3:**
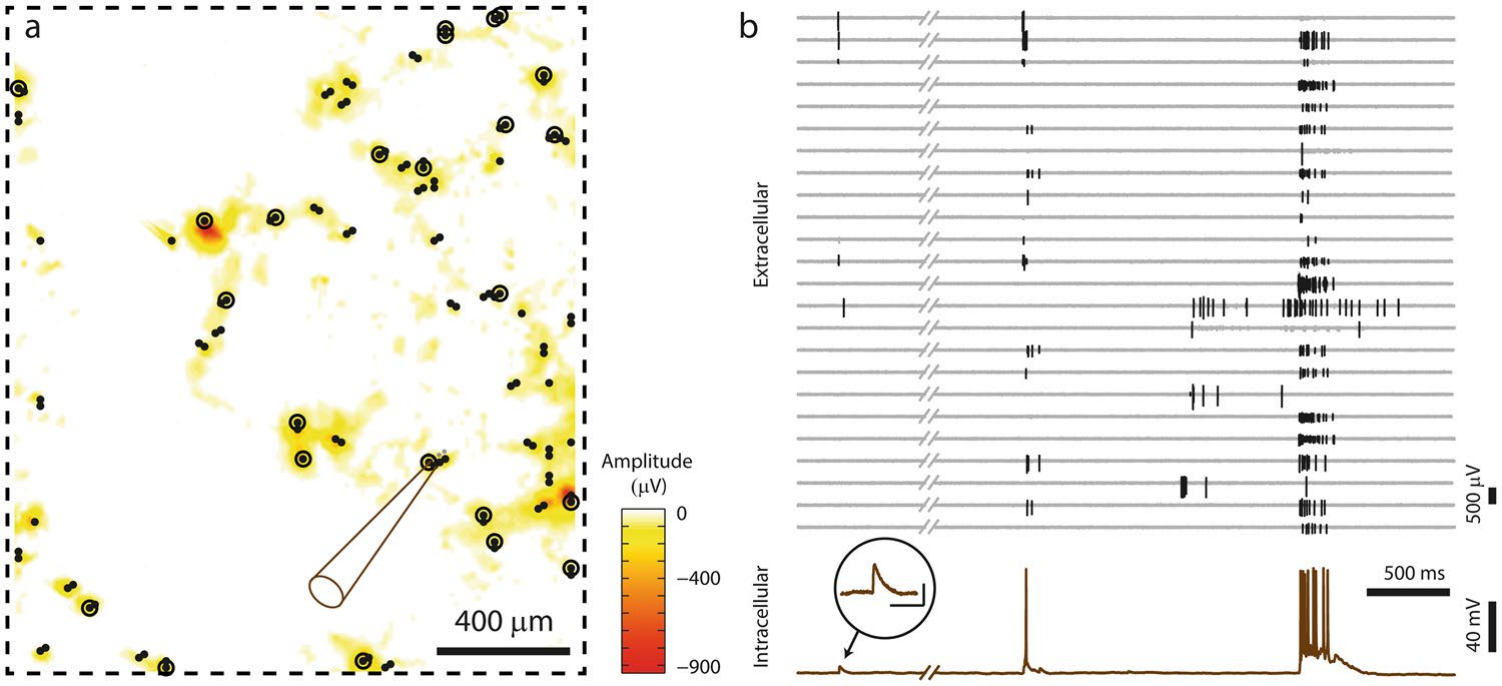
Simultaneous recording of network activity and intracellular activity. (**a**) Visualization of the array area (dashed box), and the corresponding SSAM. A neuron (position indicated by pipette drawing) was recorded intracellularly, while spontaneous extracellular activity was recorded by sparsely distributed electrodes (all recording electrodes are marked with black spots, while circled dots indicate the subset of electrodes, the signals of which are shown in (**b**)). (**b**) *Top*: Extracellular signals (detected spikes) of 24 electrodes distributed over the array at the locations of the circled dots in (**a**). *Bottom*: Intracellular signal of the patched neuron. The close-up shows an individual, enlarged PSP (scale bars 100 ms/4 mV).

The concept of measuring the intracellular activity of a patched cell, while recording extracellular signals from multiple neurons in the network, is illustrated in Fig. 3. Figure 3a shows the electrode array area (dashed rectangle) and the SSAM color-coded in the background. All electrodes selected for recording are displayed as black individual dots, and the position of the patch-clamped neuron is indicated by the sketched pipette. The *top* plot in Fig. 3b shows 24 selected extracellular signals originating from different neuronal cells distributed over the array (detected spikes), while the corresponding electrode positions are marked with black circles around the dots in Fig. 3a. At the *bottom* of Fig. 3b, the simultaneously recorded intracellular current-clamp signal of the patched neuron is displayed. As expected, correlations between the intracellular and the network activity can be observed.

These combined intra- and extracellular recordings were used in *Method 1* to identify and map individual synaptic inputs to the patched neurons. After the recording experiment, spikes in the extracellular signals were detected and sorted into neuronal units using a manually-supervised spike-sorting routine, which sorted each group of neighboring electrodes individually. We then screened the intracellular signals of the patched cell in the time windows around the spiking times of all neuronal units that have been obtained through spike sorting. Sorted units that produced significant average postsynaptic potentials (PSPs) were identified as synaptically connected to the patched cell. That the PSP was monosynaptic (direct neural connection not involving an intermediary neuron) was suggested by finding that it had constant latency and low jitter (<50 µs).

**Figure 4:**
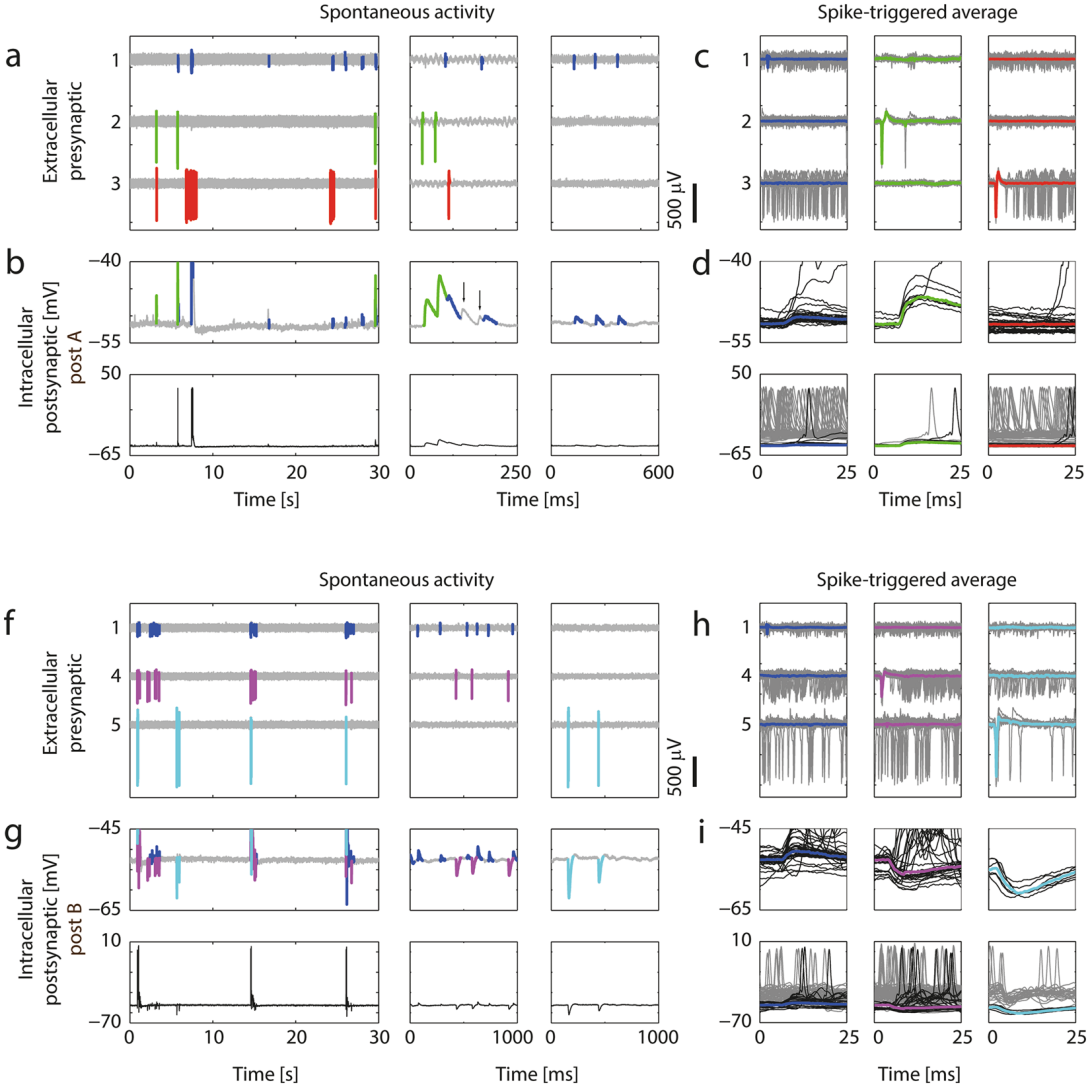
Mapping excitatory and inhibitory PSPs based on recordings of spontaneous activity. (**a**) Three data segments with different time scales (*left, center, right*) of recorded extracellular data from 3 electrodes. Spikes from 3 sorted neuronal units were colored; the blue and the green neuron were found to be presynaptically connected to the patched neuron ‘post A’, while the red neuron was not. (**b**) *Top*: Intracellular recordings from the patched cell. The signal trace after spikes of the blue and green neuron was colored in order to visualize excitatory PSPs originating from these two neurons, as determined by PSP averaging in (**d**). Note the summation of the synaptic events in the *center* plot, and that two additional EPSPs were measured, which, however, were not correlated to activity of the blue or green neurons (black arrows). *Bottom*: The same signal displayed with a larger amplitude range so that also postsynaptic APs can be seen. (**c**) STA of the extracellular APs extracted from a total of 2.5 minutes of recorded data (individual traces: gray; averaged waveforms are colored). (**d**) *Top*: Intracellular postsynaptic traces for the spikes of the colored neurons. Only traces that started from a baseline membrane potential (MP) value (i.e., did not exceed −48 mV during the first 5 ms) are shown here. Note that the blue and the green neurons evoked EPSPs of different magnitude, and that no PSPs were seen following APs of the red neuron. The colored waveforms show the median MP trace. *Bottom*: The same traces on a wider MP range. Additionally, traces which were recorded at depolarized state (MP more positive than −48 mV during the first 5 ms) are plotted in gray. (**f**–**i**) Equivalent plots for postsynaptic neuron B. In this example the violet and the cyan neurons evoked inhibitory PSPs (IPSPs) of different magnitudes, whereas the blue neuron evoked EPSPs. Note that the blue presynaptic neuron is the same than the one for ‘post A’ in Fig. 4(a–d). Spikes in h were extracted from 6 minutes of recorded data.

Figure 4 illustrates this approach for two sequentially patched neurons on the same HD-MEA chip. For patched neuron ‘post A’, we successfully identified two presynaptic neuronal units (blue and green), which caused excitatory postsynaptic potentials (EPSPs) after every presynaptic spike. Spikes of a third unit (red), which did not cause PSPs, were also included in the Figure. Figure 4a shows individual presynaptic AP spikes recorded during spontaneous activity from three selected electrodes. The corresponding intracellular signals of the patched cell are depicted in Fig. 4b and scaled accordingly to display the PSPs in the *top* and the APs in the *bottom* plots of Fig. 4b. To better visualize the mapping between presynaptic APs and PSPs, the intracellular signals immediately after spikes of the blue and green neurons were colored in Fig. 4b *top*. Figure 4b *top center* shows how individual PSPs superimpose during spontaneous activity and it shows the other PSPs (indicated by black arrows) for which the presynaptic cells were not identified.

Figure 4c shows the STA presynaptic extracellular signals, and Fig. 4d shows the resulting STA-PSPs, which represent a quantitative measure of the strength of the connection between the respective presynaptic neuron and the postsynaptic cell. During bursts of network activity, the patched cell was prevailingly in a depolarized state, which rendered it difficult to detect individual PSPs during bursting periods. Therefore, only traces that started at a membrane potential value close to the holding potential and did not exceed −48 mV during the first 5 ms, were used to calculate the average PSPs (black traces in Fig. 4d). All other intracellular traces following presynaptic spikes, for which the patched neuron was more depolarized (membrane potential > −48 mV), are colored gray in the *bottom* plots of Fig. 4d. For the blue and the red neuronal unit, the occurrence of a presynaptic AP was many times accompanied or immediately followed by a postsynaptic AP, as it can be seen by the large number of gray traces in Fig. 4d *bottom*.

**Figure 5:**
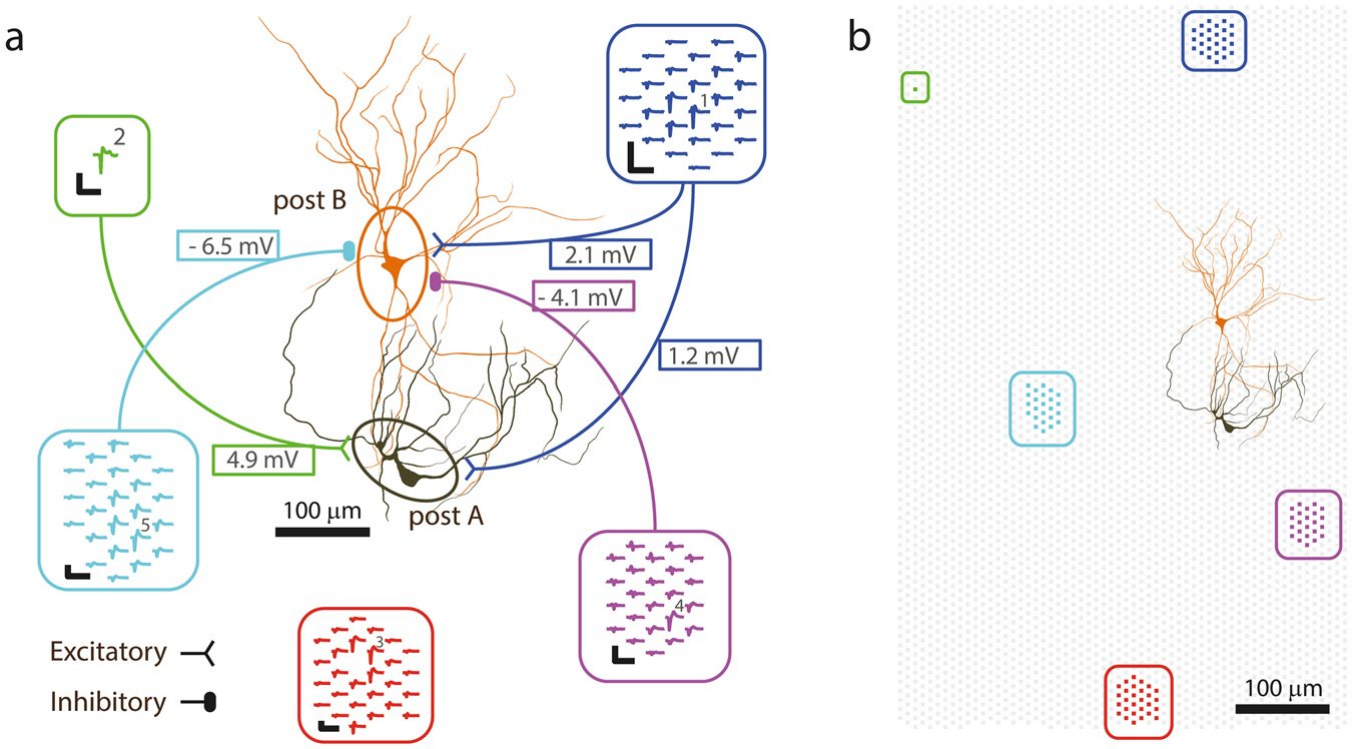
Connectivity diagram for the synaptic inputs mapped in Fig. 4. (**a**) Synaptic connections of the neurons in Fig. 4 and their average PSP amplitudes. Extracellular footprints of the presynaptic (inside rounded rectangles) and morphology of two postsynaptic neurons (in ellipses). The footprints were extracted from spontaneous activity recordings in the incubator prior to the experiment. The green neuron did not fire APs during the spontaneous scan, therefore its multi-electrode footprint could not be identified. Numbered electrodes correspond to the trace numbering in Fig. 4, scale bars of blue and violet footprint: 200 µV/3 ms; of other footprints: 400 µV/3 ms. (**b**) Locations of HD-MEA electrodes (gray background dots), electrodes used to record the footprints (colored dots) and patch-clamped neurons on the array.

Using the same PSP-mapping technique, we successfully mapped excitatory and inhibitory synaptic inputs for a second patched neuron (‘post B’). For this postsynaptic cell (Fig. 4f–i), one excitatory and two inhibitory presynaptic units were identified, where the blue excitatory presynaptic unit was the same, as the blue unit for ‘post A’ in Fig. 4a–d. Excitatory and inhibitory PSPs co-occurred spontaneously (Fig. 4g), and the STA-PSPs could be computed for the inhibitory as for the excitatory inputs (Fig. 4i). All synaptic connections mapped in this experiment are shown in the connectivity diagram in Fig. 5a. The morphology of the two postsynaptic neurons, extracted from the fluorescence imaging after patch-clamping, is shown in the center of the Figure inside the ellipses. We obtained the multi-electrode footprints of the mapped presynaptic cells by matching their waveforms with those resulting from spontaneous activity, which was previously recorded inside the incubator by using high-density electrode blocks. The relative positioning of the mapped pre- and postsynaptic neurons is shown in Fig. 5b.

### Method 2: Mapping Stimulation-triggered PSPs

The number of mapped synaptic connections for *Method 1* strongly depends on the characteristics of the spontaneous activity. Presynaptic neurons with tonic firing can be mapped more easily, whereas neurons featuring a high correlation of their activity to that of the postsynaptic neuron or other neurons are difficult to map. Since the HD-MEA allows for applying precise and confined electrical stimuli at any selected electrode, we sought to increase the number of mapped synaptic connections through stimulation-based PSP-mapping strategies.

A target neuron can be affected by extracellular HD-MEA stimulation in two ways. The neuron is either directly activated by the electrical stimulus (direct activation), which causes a change in membrane potential and possibly evokes APs. A second possibility is presynaptic activation, where an AP is evoked at a presynaptic neuron, which then leads to a PSP and possibly to a postsynaptic AP at the target cell. Here, we used the combined HD-MEA and patch-clamp setup to demonstrate and characterize both, direct and presynaptic - excitatory and inhibitory - activation of neurons upon extracellular HD-MEA stimulation.

**Figure 6:**
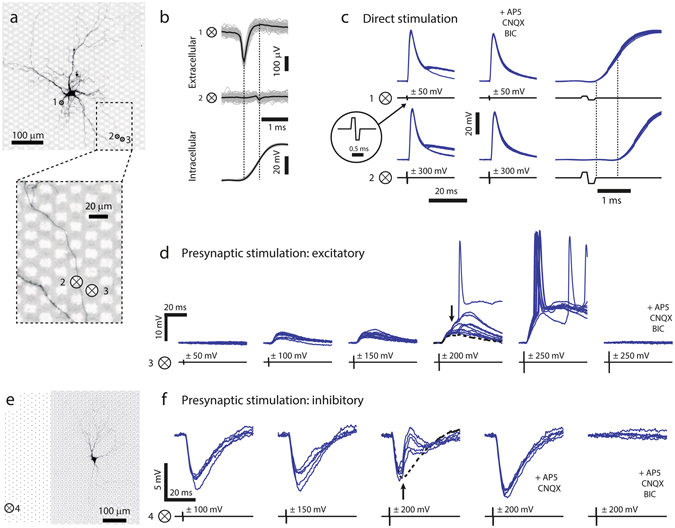
Evoking postsynaptic signals through HD-MEA electrical stimulation. (**a**) Fluorescence image of a neuron patched on the array, the numbered crosses inside circles “⊗” indicate positions of stimulating and/or recording electrodes. The magnified part at the *bottom* shows that a neuronal process is situated on top of *electrode 2*. (**b**–**e**) Numbered ⊗ correspond to the electrodes indicated in a. (**b**) STA extracellular traces (gray) and average trace (black), measured at *electrodes 1* and *2* (*top*) and intracellularly measured AP (*bottom*). Note differences in spike shape and the temporal delay between the waveforms at *electrodes 1* and *2*. (**c**) *Left:* Intracellular responses (blue traces, 10 trials each) to extracellular stimulation at *electrodes 1* and *2* under control conditions. The stimulus timing is indicated by the black traces, and the inset shows a single biphasic voltage pulse. All stimuli evoked APs, some stimulation trials also evoked PSPs, which overlapped with the APs. *Center*: Responses in the presence of synaptic blockers, where no PSPs were evoked. *Right*: Zoom-in on responses under control conditions to better visualize the delay between stimulus end and AP onsets (depicted by the dashed lines). Also note the difference in stimulus amplitude required to elicit an action potential through *electrodes 1* and *2*. (**d**) Stimulation with voltage pulses of increasing amplitudes. APs of a presynaptic neuron were evoked with stimulation voltages of ±100 and ±150 mV leading to PSPs. Increasing the voltage to ±200 mV resulted in additional PSPs for some trials (indicated by the black arrow, the dashed black line represents the median response for ±150 mV pulses). Yet larger PSPs were seen for ±250 mV, along with the occurrence of postsynaptic APs in most of the trials. Addition of synaptic blockers resulted in complete blockade of evoked signals, indicating that all observed responses involved synaptic transmission. (**e**) Fluorescence image of another neuron and position of the stimulation *electrode 4*. Black dots represent the positions of MEA electrodes. (**f**) PSPs upon stimulating an inhibitory presynaptic neuron were evoked by applying ±100 and ±150 mV at *electrode 4*. An additional excitatory presynaptic neuron was evoked upon increasing the extracellular stimulus at *electrode 4* to ±200 mV (black arrow, black dashed line represents the median response for ±150 mV). Application of excitatory blockers blocked the EPSPs at ±200 mV, whereas additional application of BIC completely blocked all responses.

Figure 6a shows the image of a patched neuron and the position of three selected electrodes (circled, black crosses) which were used for electrical recording and stimulation. After break-in, the footprint and the cell outline were obtained as described above. The individual (gray) and STA (black) extracellular traces recorded at *electrode 1*, close to the soma, and at *electrode 2*, located at a distal neuronal process (Fig. 6a *close-up*), are shown in Fig. 6b, together with the average intracellular trace at the *bottom*. The spike at *electrode 2* (located 200 µm away from the cell body) shows typical features of axonal APs^28, 31^: small amplitude, positive-first triphasic spike shape and significant delay (0.55 ms) with respect to the STA extracellular AP near the soma at *electrode 1*.

We reliably evoked APs in the patched cell by applying biphasic voltage pulses at *electrode 1* (±50 mV) or at *electrode 2* (±300 mV). Figure 6c *left* shows the intracellular signals in response to ten HD-MEA stimulation trials under control conditions. In both cases, some stimulation trials also triggered APs in one or multiple presynaptic cells that, in turn, evoked PSPs in the patched cell, which then overlapped with the observed APs. The addition of synaptic blockers (APV, CNQX and BIC) to the perfusion medium entirely eliminated the postsynaptic signals but did not affect the APs at the patched neuron (Fig. 6c *center*). Whereas stimulation at *electrode 1* evoked APs close to the cell, stimulation at *electrode 2* evoked an AP in the axon, which then propagated antidromically to the cell body. For this reason, the timing of the intracellular AP onset upon stimulation at *electrode 2* was significantly delayed as compared to the intracellular AP timing for stimulation at *electrode 1* (*right* plot of Fig. 6c). The antidromic propagation delay (dashed lines in Fig. 6c) and the orthodromic signal propagation time until reaching the axonal position of *electrode 2* (dashed lines in Fig. 6b) have comparable length (0.63 ms and 0.55 ms respectively).

Both examples in Fig. 6c, stimulation at the axon and stimulation next to the soma, represent modes, where the target cell is directly activated by the stimulus. Besides the direct activation and combined direct and presynaptic activation, as shown in Fig. 6c *left*, pure presynaptic activation of target neurons was also frequently achieved. This stimulation type is illustrated in two examples in Fig. 6d–f, where excitatory and inhibitory postsynaptic signals, evoked by HD-MEA stimulation, were detected.

Figure 6d shows the intracellular signals of the patched cell in Fig. 6a in response to stimuli applied at *electrode 3* with variable stimulation voltage (ten individual trials per stimulus). Stimulation through a ±50 mV pulse did not evoke any response at the patched neuron, whereas single and presumably monosynaptic PSPs were measured for stimuli of ±100 mV or ±150 mV. When the stimulation voltage was further increased to ±200 mV, some trials showed postsynaptic signals with larger amplitudes (black arrow in Fig. 6d), indicating that multiple presynaptic cells were simultaneously activated. Stimulation pulses of ±250 mV finally evoked additional presynaptic sites, resulting in suprathreshold synaptic signals, which caused the postsynaptic neuron to fire APs for most trials. All evoked signals were mediated by synaptic transmission, as they were completely suppressed upon addition of synaptic blockers (APV, CNQX and BIC).

For a different patched neuron, visualized in Fig. 6e, inhibitory postsynaptic signals upon HD-MEA stimulation were measured, if extracellular stimulations were applied at *electrode 4*. Stimulation pulses with ±100 mV and ±150 mV reliably evoked inhibitory PSPs (IPSPs), as shown in Fig. 6f (five individual trials per stimulus). Increasing the stimulation voltage to ±200 mV additionally activated a second, excitatory, presynaptic neuron that evoked EPSPs, which were superimposed to the IPSPs (indicated by the black arrow). The EPSPs were suppressed upon adding APV and CNQX (AMPA and NMDA receptor antagonists) to the bath solution. Subsequent addition of BIC, a selective GABA_A_ antagonist, also blocked the initial IPSPs.

Based on the presented measurements, a suitable strategy to identify stimulation-triggered, monosynaptic PSPs from individual neurons includes to stimulate with variable voltages at multiple electrodes, and to then select the lowest stimulation voltage, for which PSPs are reliably evoked. Stimulation, however, should be applied in the vicinity of neurons, in order to achieve a high stimulation efficiency and low stimulation thresholds. For this reason, stimulation electrodes were selected to feature large recorded spike amplitudes in the SSAM (see *Methods*).

**Figure 7:**
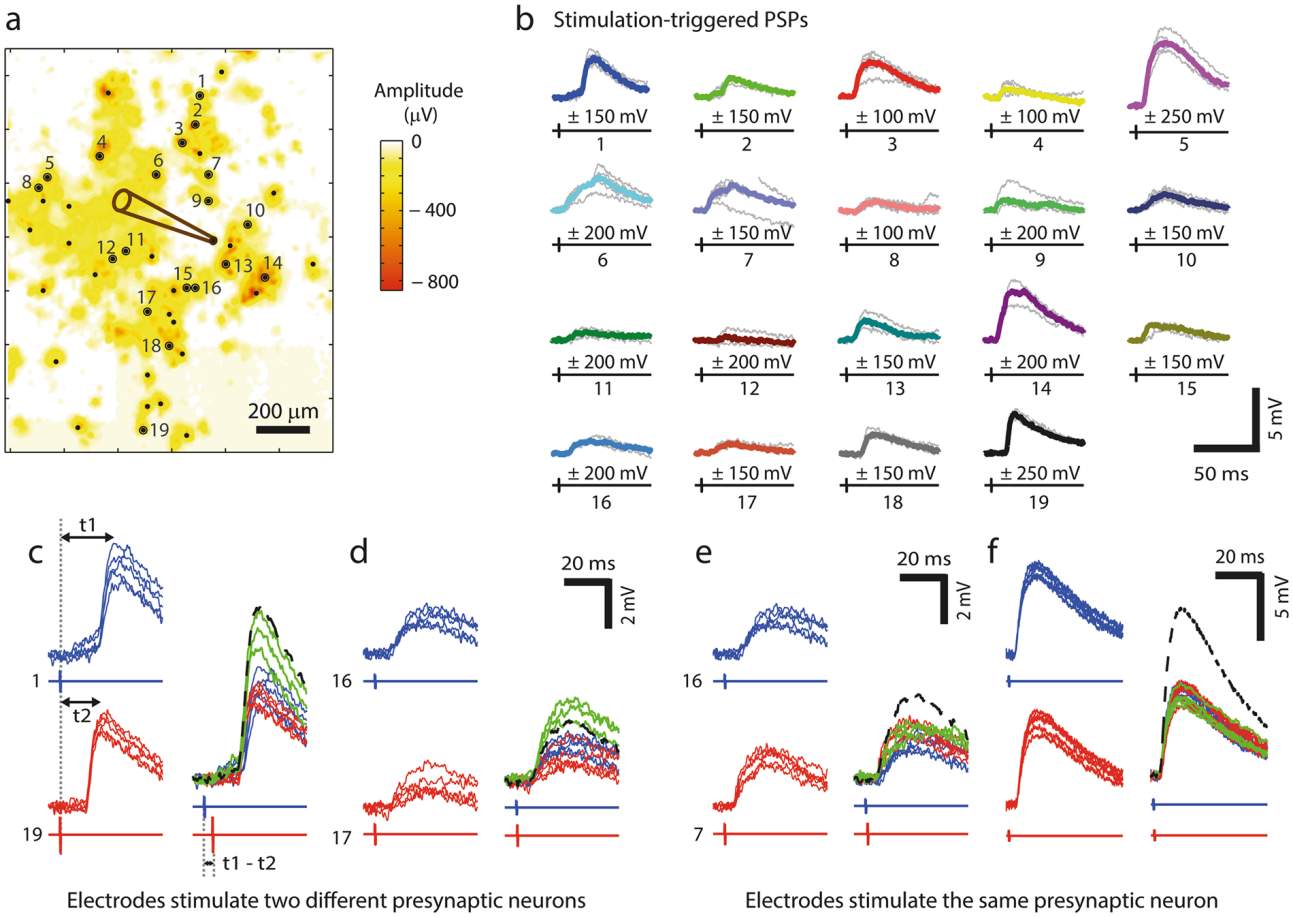
Stimulation-triggered PSPs from multiple presynaptic inputs. (**a**) SSAM of a MEA region with the color code indicating negative peak amplitudes of the spikes at each electrode. The position of the patched cell is indicated by the pipette drawing. Electrode locations (depicted as black dots) were selected according to large negative peaks in the recorded signal amplitude map and then used for stimulation with bipolar voltage pulses of 100, 150, 200 and 250 mV amplitude. Small dots mark electrodes that either did not evoke any PSP response or could not be associated with individual monosynaptic PSPs; large dots mark electrodes through which the stimulation yielded individual PSPs. (**b**) PSP responses (gray: individual traces; median traces are colored) for stimulation through the numbered electrodes in (**a**), where the lowest PSP-evoking stimulation voltage was chosen for every electrode. The stimulus timing are visualized by the black signals below the PSPs. (**c**) *Left*: PSPs and stimulus for *electrodes 1* and *19* (from **b**), which exhibited similar amplitudes but different latencies (t1, t2: time between stimulation pulse and PSP maximum). *Right*: Paired stimulation for *electrodes 1* and *19*, where the timing between the two stimuli was t1 – t2 = 3.65 ms. The green traces show responses to paired stimulation, and red respectively blue traces represent the PSP responses to the individual stimuli (as shown *left*). The green responses to paired stimulation featured clearly larger amplitudes than the responses to individual stimuli, suggesting that the PSPs obtained through stimulation at *electrodes 1* and *19* originated from two different presynaptic inputs. The black dashed line visualizes the theoretical sum of the individual average PSP responses. (**d**) Second example showing significantly larger responses to paired stimulation as compared to individual stimulation, thus indicating two different presynaptic sources. (**e**) Example, where paired stimulation does not produce larger PSP amplitudes than individual stimuli, indicating that stimulation of electrodes 16 and 7 activated the same presynaptic neuron. Note the comparably large spatial distance between the two electrodes in (**a**). (**f**) Example from another dataset, where PSP responses upon stimulation through two different electrodes were also evoked by the same presynaptic neuron.

Figure 7 shows the SSAM for such an experiment, and all selected electrodes were marked by black dots. We applied bipolar voltage stimuli of ±100 mV, ±150 mV, ±200 mV and ±250 mV amplitude (5 trials per voltage) in a fully randomized order. On a subset of the electrodes, the stimuli evoked EPSPs in the patched neuron. We then manually identified for each of these electrodes the corresponding lowest stimulation voltage, which reliably yielded EPSPs in the patched neuron. Figure 7b shows the individual (gray) and average (colored) EPSP traces for the electrodes (numbered black dots in Fig. 7a) obtained with this approach. The high temporal precision of the EPSPs indicated that they originated from individual stimulated presynaptic neurons featuring a monosynaptic connection to the patched cell. Accordingly, the electrodes and stimulation voltage values obtained with this approach and the possibility to individually stimulate a large number of presynaptic cells enable to precisely control multi-neuronal synaptic input to the patched cell.

The number of synaptic connections mapped with this approach is summarized in Table 1 for eight patched cells. Postsynaptic signals in response to HD-MEA stimulation were manually classified into the categories *no signal evoked*, *single PSP evoked, multiple PSPs evoked, AP evoked*. *Single PSP evoked* was selected if, one PSP was evoked upon applying a range of stimulation voltages (as in Fig. 6d for ±100 mV), whereas, if the shape of the postsynaptic signals presumably originated from multiple PSPs (as in Fig. 6d for ±200 mV), the result was assigned to the category *multiple PSPs evoked*. *AP evoked* was selected if the stimulation was followed by an AP at the patched cell, presumably evoked by direct stimulation (as in Fig. 6c). The summary in Table 1 illustrates the variations in connectivity between the different cell cultures and patched neurons.

**Table 1:** Mapped connections upon randomized HD-MEA stimulation for eight patched cells.

Patched Neuron no.	Stimulated electrodes	No signal evoked	Single PSP evoked	Multiple PSPs evoked	AP evoked
1	15	11	0	0	4
2	51	34	14	0	3
3	24	21	0	1	2
4	39	36	1	1	1
5	60	29	22	9	0
6	44	13	19	12	0
7	44	30	6	6	2
8	44	18	12	11	3

Number of synaptic connections for eight postsynaptic neurons, where *Neurons 6, 7*, and *8* were patched in the same culture.

As we have shown in Fig. 6, neurons could be stimulated at different sites close to the soma and along the axon. Furthermore, axonal outgrowth can extend over large distances across the array^28^. Therefore, it is reasonable to assume that specific presynaptic neurons were stimulated multiple times through different stimulation electrodes in Fig. 7b. As a consequence, not all PSPs originated from different presynaptic cells. For a wide range of experiments, however, it is necessary to evoke and control PSPs, which originate from specific and different presynaptic cells. We therefore applied a paired presynaptic stimulation protocol^10^ to test, whether PSPs with similar amplitudes were caused by stimulating the same presynaptic neuron through different electrodes.

PSPs with similar amplitudes were evoked in combination, and the individual stimulation timings were adjusted so that the maxima of both PSP traces would occur simultaneously (Fig. 7c). In cases where PSPs originated from different presynaptic cells, the evoked PSPs summed up, and the measured postsynaptic signals resulting from paired presynaptic stimulation were significantly larger in amplitude than the PSP signals evoked by stimulating only one presynaptic cell (synaptic inputs in Fig. 7c,d). In contrast, Fig. 7e,f show two examples, where the PSPs evoked through stimulating two different electrodes originated from the same presynaptic neuron. Note that such stimulations of the same presynaptic neuron by two electrodes were also found for electrodes with large spatial distances, as in the case of electrodes *7* and *16* being 420 µm distant.

### Plasticity measurements from multiple synaptic inputs

We have presented a method to assign and evoke stimulation-triggered PSPs by combining a HD-MEA with a patch-clamp setup. Once suitable stimulation electrodes and the corresponding stimulation voltages were identified, synaptic events could be arbitrarily triggered at the patched cell at microsecond temporal precision. In the following, we demonstrate the capabilities of this setup to measure basic characteristics of short- and long term synaptic plasticity upon applying a paired-pulse protocol and intracellular tetanization (IT). The corresponding measurements were performed by activating multiple synaptic inputs of a single, patched cell.

**Figure 8:**
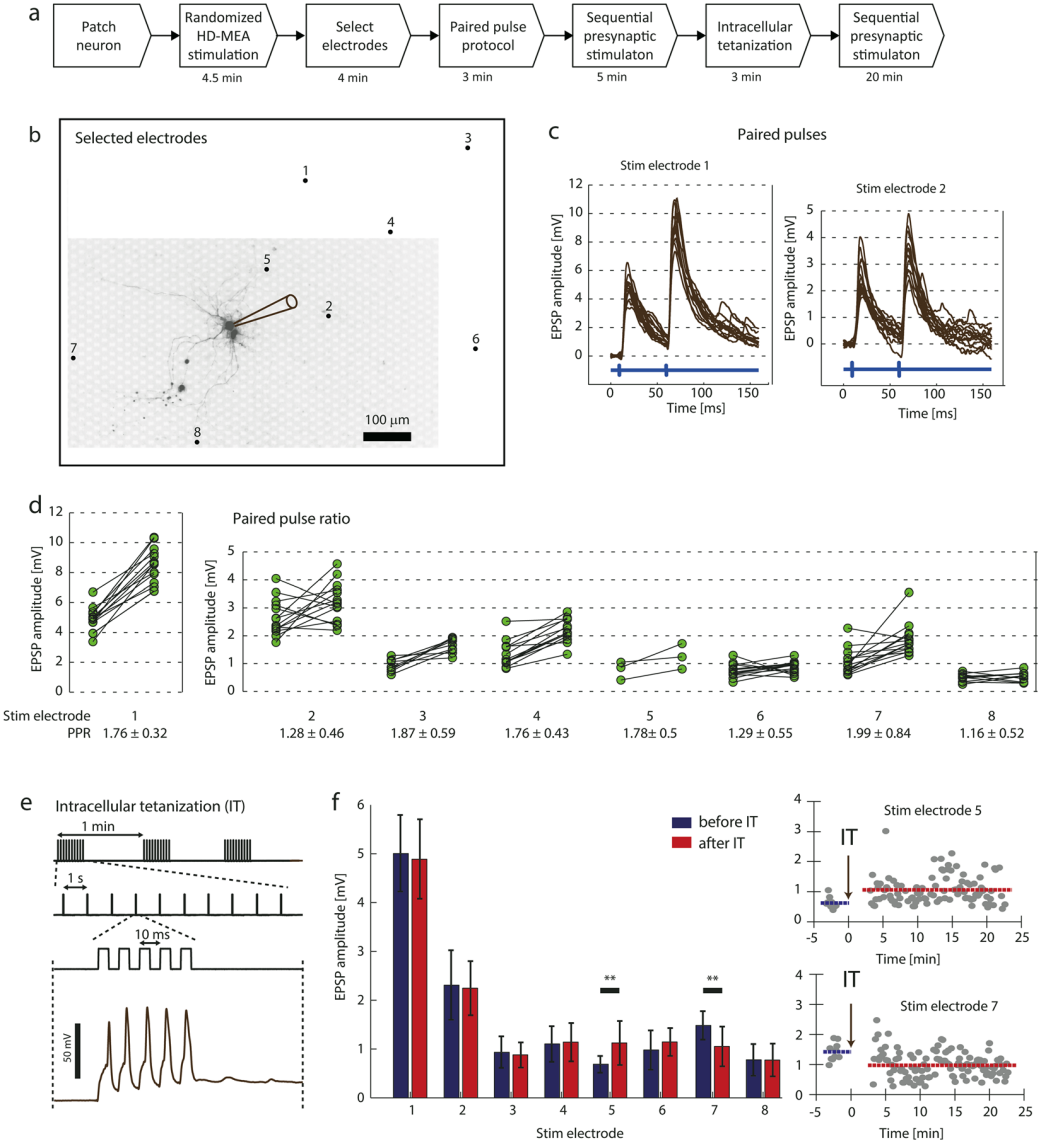
Short- and long-term plasticity measurements for multiple synaptic inputs. (**a**) Experimental steps. (**b**) Fluorescence image of a patched neuron (indicated by the pipette drawing) and position of eight electrodes, which were found to evoke PSPs at the patched cell. (**c**) Individual intracellular traces during application of paired-pulse stimulation through *SE 1* and *SE 2* (14 trials each). The blue signal at the *bottom* visualizes the timing of the extracellular stimulation. (**d**) Every connected pair of dots displays the PSP amplitude values for the first and the second evoked PSP upon application of a paired-pulse protocol. If the postsynaptic neuron depolarized before the second PSP peak occurred, the PSP values could not be measured and the trial was ignored for the Figure and PPR calculation. (**e**) *Top*: Current stimulus used for the intracellular tetanization protocol as described in *Methods*, consisting of three trains with 10 individual bursts while each burst included five current pulses (0.9 mA amplitude, 5 ms duration) evoking individual APs. *Bottom*: Five APs evoked by a burst. (**f**) *Left*: Average PSP amplitudes before and after IT and the corresponding standard deviations indicated by error bars. *Right:* Individual measured PSP values over the course of the IT experiment for *SE 5* and *SE 7*. The blue and red lines visualize the average PSP amplitude before and after IT.

The individual experimental steps are visualized in Fig. 8a. A neuron was patched on top of the array, and eight electrodes, which reliably evoked PSPs at the patched cell were selected by using the randomized HD-MEA stimulation protocol (see *Methods*). The patched cell and the positions of the selected electrodes are shown in Fig. 8b. Next, a paired-pulse protocol (PPP) was executed. It consisted of pairs of stimulations at 50 ms interval, which were applied sequentially through the selected electrodes to measure the paired-pulse ratio (PPR). Figure 8c shows the intracellular traces during pairs of stimulations for two stimulation electrodes (SEs). The green connected dots in Fig. 8d represent the individual amplitude values for the first, respectively the second PSP of a pair. Overall, five synaptic inputs (activated through stimulation of electrodes 1, 3, 4, 5, 7) showed considerable short-term facilitation with PPR values larger than 1.5. *SE 1* is an example, where the second PSP was always significantly larger with an average PPR of 1.76. For *SE 2*, on the other hand, we found irregular PSP amplitudes in response to the PPP, which indicates both, facilitation and depression of the second peak during the trials.

We then examined the effect of intracellular tetanization (IT) on the synaptic inputs. IT is a purely postsynaptic protocol^11, 32^, which has been shown to cause plastic changes in synaptic connections over timescales of tens of minutes. First, we measured the synaptic connections by evoking PSPs at 1 Hz sequentially on the selected 8 electrodes during five minutes. We then applied the IT protocol, depicted in Fig. 8e, to the patched cell. The protocol consisted of three trains of 10 bursts each, with five APs evoked at high-frequency (100 Hz) per burst. Finally, PSPs were again measured for another 20 minutes by sequentially stimulating the selected 8 electrodes (1 Hz).

The average PSP amplitude values before and after IT are illustrated in Fig. 8f *left*. While the amplitude of most PSPs did not substantially change after IT, we found significant differences between the average PSP amplitude before and after IT for *SE 5* (two-sample *t*-test, *p* = 0.0038) and *SE 7* (*p* = 0.0019). The individual PSP amplitude values over the course of the experiment for these two cases are plotted in Fig. 8e at the *right*. The PSPs evoked by stimulating *SE 5* increased after IT and showed a long-term potentiation (LTP) effect. On the other hand, long-term depression (LTD) was observed at the PSPs evoked by stimulating *SE 7*. Interestingly, IT can induce both LTP and LTD at two inputs to the same cell, which has also been reported previously by Chen *et al*.^11^.

## Discussion

The major advance in this work includes the combination of intracellular and high-resolution extracellular microelectrode-array-based electrophysiology. In previous studies, a combination of patch-clamp with conventional, low-resolution MEA technology has been used to correlate intracellular single-cell and network-level spiking activity^33^ and to evoke network bursts^34^ and monosynaptic PSPs at the patched cell^35^ through MEA stimulation. In contrast to conventional MEAs, HD-MEA technology allows for recording from and stimulating of individual, identified neurons, which enables to study details of the computation of individual neurons within a network context and to resolve individual synaptic inputs.

We developed a set-up that allowed us to patch cultured neurons directly on top of HD-MEAs under visual supervision, and we describe two methods for identifying multiple synaptic inputs to individual neurons in culture. The presented experimental procedure is comparably simple - only one neuron needs to be patched at a time. By combining the acquisition of the intracellular and extracellular signals at hardware level, the system allows for fast and efficient data processing during the experiment, in order to, e.g., scan and identify synaptic connections within a short time. In contrast to optical methods, the HD-MEA-based approach allows for detecting and evoking APs at microsecond resolution and for accurately averaging the resulting PSPs. Furthermore, the experiments do not require any special dyes, chemicals compounds or the uncaging of agents, which might entail side-effects, such as blockage of receptors, phototoxicity effects, or have an influence on intracellular calcium dynamics.

### Measuring neural connectivity

In *Method 1*, the HD-MEA was used to record the spiking activity of multiple cells, while individual neurons were recorded intracellularly. This method requires accurate spike sorting in order to identify the synaptic inputs. Neighboring electrodes were selected at the recording sites, so that every cell was recorded by multiple electrodes. This spatial sampling is an important factor for spike sorting, especially due to challenges posed by overlapping spikes and spike waveform variability during bursts^36, 37^. Note that, in the present work, offline spike sorting was performed after the experiment. Nevertheless, the HD-MEA provides a suitable platform for online spike sorting by using, e.g., template-matching techniques^37–40^. The identification of the templates could be performed while the culture is still kept under incubation conditions, and online spike sorting would then allow for immediate identification of synaptic inputs during the recordings.

A similar approach for synaptic mapping, based on measuring spontaneous presynaptic APs, was previously described for using calcium imaging and patch clamp recordings^15, 16^. This technique makes use of a reverse-correlation analysis to identify neurons that fire APs time-locked with detected synaptic events and has proven to be powerful to identify synaptic projections^41^. However, detecting of the individual presynaptic APs is very challenging due to limitations in the SNR and the temporal resolution of the calcium signals.

In contrast, our approach allows for measuring temporally-precise, single, presynaptic APs and is, therefore, suitable to identify monosynaptic connections (Fig. 4). Furthermore, temporal features, such as synaptic latency, can be measured and analyzed in a multi-neuronal context. Since no stimulation is applied, the experiment does not actively influence the intrinsic network activity. It is therefore possible to measure and analyze synaptic effects during spontaneous activity, such as changes in PSP latency, PSP strength or PSP summation characteristics (Fig. 4b). Neurons that have been identified during an experiment can also, based on their footprints, be matched with extracellular data that was previously recorded. We applied this technique in Fig. 5a, where the footprints of the identified presynaptic neurons were identified based on previously recorded spontaneous activity. Since cultured neurons on HD-MEAs can be recorded from for extended periods of time up to several months^42–44^, a combination of synaptic mapping with long-term HD-MEA recordings would allow for gaining access to the spiking history of mapped cells and to analyze them in a long-term context of network activity.

A difficulty of PSP-mapping, based on spontaneous activity, is the requirement of spontaneous, non-synchronous neuronal activity. Typically, cultures of dissociated cortical neurons oftentimes show a high degree of synchrony and collective bursting activity. During network bursts, the postsynaptic cell is mostly in a depolarized state so that individual PSPs cannot be properly measured. A possible approach to overcome this problem would be a local puff application of glutamate through a glass pipette to evoke nonsynchronized spikes of presynaptic candidate neurons^16, 45^. Also, using the voltage-clamp technique instead of current-clamp would reduce this problem, as depolarization of the clamped cell is then prevented.

### Multisynaptic integration

Using the second method for synaptic mapping presented here, neuronal APs were evoked by extracellular voltage stimulation through the HD-MEA electrodes, while the intracellular signal of individual neurons was recorded. As opposed to *Method 1*, this approach does not require particular spontaneous neuronal activity. Stimulation-based synaptic mapping has been previously performed with different stimulation techniques: Electrical stimulation through individual bipolar electrodes^11, 12^ or stimulation arrays^10, 46^, as well as optical stimulation by using glutamate uncaging^17, 47, 48^.

Approaches based on electrical stimulation have the advantage that they deliver temporally precise and reproducible stimuli, however, they usually are compromised in spatial resolution. For this reason, the number and selectivity of activated presynaptic cells may be limited and largely depends on number, dimension and location of the extracellular electrodes. Optical stimulation with glutamate uncaging, on the other hand, offers high spatial resolution and allows for stimulating many cells, even across different focal planes^48^. A dynamical control of the light beam has furthermore allowed to generate spatio-temporally structured synaptic input patterns^9, 47^.

However, the latter technique also has some substantial limitations. Direct stimulation of the postsynaptic cell must be avoided, and the laser power and pulse duration must be carefully calibrated. Still, evoking single presynaptic APs is difficult to reliably achieve, and the temporal resolution is limited. Furthermore, caged neurotransmitters were found to block GABA_A_ receptors to some extent^49, 50^, which can lead to epileptiform events and prevents the study of inhibitory synaptic transmission. Due to these limitations, the method is not suitable for short- and long-term plasticity experiments^51^.

Featuring 11,011 electrodes that can be individually selected for stimulation, our HD-MEA provides the possibility to activate hundreds of neurons during a synaptic mapping experiment. The main limiting factor is the temporal duration of the stimulation protocol with respect to the time a neuron can be patch-clamped. The stimulation protocol depends on the experimental parameters stimulation frequency, number of electrodes, number of voltages applied and stimulation trials. In the experiment shown in Fig. 8, for example, 8 synaptic inputs were found within 4.3 minutes, while 52 electrodes were probed with 5 stimulation voltages and 4 trials each at 4 Hz. Assuming that the stimulation frequency can be safely increased to 10 Hz, while the other parameters are kept, 400 electrodes could be probed within 13 minutes. In contrast to uncaging techniques, HD-MEA stimulation does not have side-effects, such as the blockade of inhibitory receptors. Therefore, excitatory and inhibitory signals can be concurrently measured, as shown in Figs 4 and 5.

In Fig. 8, we demonstrated basic plasticity experiments, where the PPRs and the PSP amplitudes before and after postsynaptic tetanization were measured. Having the possibility to identify multiple synaptic inputs and to precisely trigger PSPs in defined patterns, our system allows for conducting a wide range of electrophysiological experiments. For example, patterns of presynaptic activity could be activated to simulate activity of neuronal ensembles and to measure their effects on the individual synaptic inputs. Also, spike-timing dependent plasticity experiments for multiple presynaptic cells can be performed by precisely controlling pre- and postsynaptic APs.

Finally we would like to mention another field of application, which is heterosynaptic plasticity^10, 11^. Here, plasticity at activated synapses through presynaptic stimulation has been shown to modulate opposite changes in other, non-activated synapses. This mechanism is thought to prevent the postsynaptic cell from overexcitation after intense synaptic potentiation. Our system can be used to induce plasticity at individual synapses by applying tetanic stimulation of presynaptic cells, or by inducing spike-timing dependent plasticity through combined pre- and postsynaptic stimulation at controllable delays. Effects of induced plasticity on other, nonactivated synapses could be then characterized with large sample size. In general, the presented system could be valuable to any application, where interactions between multiple synaptic inputs are of interest.

## Methods

### HD-MEA system

We used a CMOS-based HD-MEA system^22, 30^ for extracellular neuronal recording and stimulation. The electrode array was integrated into a microsystem chip, which has been fabricated in a 0.6-μm CMOS process; the chip accommodates a total of 11,011 electrodes in an area of 1.99 × 1.75 mm^2^ (17.8 μm center-to-center pitch, 3′161 electrodes/mm^2^ density, 8.2 × 5.8 µm^2^ electrode size). A subset of 126 electrodes can be simultaneously recorded from, by connecting those by means of a flexible switch matrix, located underneath the array, to the 126 read-out channels. The switch-matrix approach provided low-noise (2.4 μV_rms_) recordings and high routing flexibility to select almost arbitrary electrode configurations. Furthermore, each electrode can be connected to one out of two stimulation channels for voltage or current stimulation^52^ so that it can be stimulated with arbitrary waveforms, which are provided by two individual digital-to-analog converter units.

The recorded signals were amplified (57 dB), low-pass filtered (14 kHz) and digitalized (8 bit, 20 kHz) on-chip, and sent to a field-programmable gate array (FPGA) board. The data were then streamed to a Linux-based host PC for data storage and real-time visualization with an adapted version of the MeaBench software^53^. MATLAB (The Mathworks) was used for data analysis and visualization during the experiments, as well as for controlling and sending commands to the chip (i.e., for electrode selection, recording and stimulation protocols).

In order to effectively reduce the electrode impedance and to improve recording and stimulation conditions by increasing the effective electrode surface area, platinum black was deposited on the HD-MEA electrodes. A current of 180 µA was simultaneously applied to all electrodes for 45–75 s, and a platinum wire to be used as a ground electrode was immersed in the deposition solution (7 mM hexachloroplatinic acid and 0.3 mM lead (II) acetate anhydrous). Deposition uniformity was improved by wiping the platinum black from the electrode area with a cotton stick and repeating the procedure 1 or 2 times.

### Setup for combined HD-MEA and Patch Clamp Recordings

The experimental set-up is illustrated in Fig. 1 and combined the HD-MEA system with an upright microscope (Leica DM6000B) and a conventional patch clamp system. The HD-MEA chip and the patch clamp micromanipulator (Sutter Instruments) were positioned on a fixed stage (Scientifica), whereas the microscope was mounted on a motorized XY stage (Scientifica UMS), which allowed for imaging a large area. The imaging software (Leica LAS AF) on the Windows workstation controlled the microscope as well as the XY stage position through the microscope controller (CTR7000 HS). The precise XY position of the microscope was stored for every acquired image. The acquired images were automatically aligned with the corresponding HD-MEA coordinates by using custom image alignment software (MATLAB) on the Linux workstation. This feature was particularly important to identify the electrodes underneath a patched cell, or to localize a particular neuron based on the activity measurements with the HD-MEA.

To record the signals from the patch clamp amplifier, the FPGA of the HD-MEA setup was equipped with four analog-to-digital conversion channels (ADCs, AD974 Analog Devices) on a custom printed-circuit board. The ADCs provided 16 bit conversion for a ±10 V input range and were synchronized to the HD-MEA sampling rate. On the FPGA, the digitalized signals were inserted into the HD-MEA data stream, which was sent to the Linux workstation and stored. The patch clamp signals could be visualized together with the HD-MEA signals in real-time (MeaBench), which was crucial for correlating intra- and extracellular measurements during the experiment.

### Cortical neuron culture preparation

Embryonic-day 18 Wistar rat cortices were dissociated in 2 ml trypsin with 0.25% EDTA (Life Technologies, Bleiswijk, Netherlands) with trituration. The array was pre-coated with a thin layer of poly(ethyleneimine) (Sigma, Buchs, Switzerland), 0.05% weight/weight, in borate buffer (Chemie Brunschwig, Basel, Switzerland) at a pH of 8.5, followed by a drop of 0.02 mg ml^−1^ laminin (Sigma) in Neurobasal (Life Technologies). 15000–20000 cells in a 30 µl drop were seeded over the array. 1 ml of Neurobasal medium was added after 30 minutes. The cultures were maintained inside an incubator under controlled environmental conditions (37 °C, 65% humidity, 5% CO_2_) in 1 ml of Neurobasal medium, which was partially replaced twice per week (50%).

Including the experiments to establish the methods and to develop the experimental protocols, a total of 30 cultures were used in which 40 neurons were patched. Spontaneous presynaptic activity was recorded for 6 patched cells (*Method 1*) and synaptic mapping through randomized MEA stimulation was applied to 8 neurons (*Method 2*). Five neurons in three cultures were patched for testing and evaluating the plasticity protocols.

### Patch Clamp Electrophysiology

Patch clamp experiments were performed after 2–5 weeks in culture. For the patch clamp experiments, the cultivation medium was removed, and the chip was perfused with a HEPES-buffered external bath solution containing (in mM:) NaCl 149, KCl 3.25, CaCl_2_ 2, MgCl_2_ 2, HEPES 10, Glucose 11 (pH: 7.35 adjusted by using NaOH 1 M). The bath was constantly perfused during the experiment at a low rate, and all experiments were performed at room temperature. Neurons on top of the MEA electrodes were visualized in bright-field mode by using difference interference contrast (DIC) optics of the upright microscope. We found that the deposited platinum black reduced the strong contrast between the electrodes and the passivated chip surface by darkening the array electrode surfaces, which resulted in greatly improved visibility of the cells. The micropipettes had resistances of 5–7 MΩ and were filled with an internal solution containing (in mM): C_6_H_11_KO_7_ 135, KCl 20, MgCl_2._6H_2_O 2, HEPES 10, EGTA 0.1 Na_2_ATP 2, Na_3_GTP 0.3, adjusted to a pH of 7.3 with KOH. In most cases, 0.02 mM Alexa Fluor 594 (Life Technologies) was added to the internal solution, and fluorescence images were acquired during and after the patch clamp experiment. The cells reported in this paper had holding potentials below −50 mV, and the junction potential was not corrected for. All recordings shown in this study were performed in the current clamp mode, and the patch clamp amplifier was controlled through the open-source software WinWCP (John Dempster, University of Strathclyde, UK).

To block excitatory synaptic activity, 100 µM of the AMPA antagonist 6-cyano-7-nitroquinoxaline-2 3-dione (CNQX), and 10 µM of the selective NMDA receptor antagonist DL-2-amino-5-phosphonovaleric acid (AP5) were added to the bath solution. Addition of 50 µM Bicuculline methiodide (BIC), a GABA_A_ antagonist, was used to block inhibitory synaptic signaling.

### HD-MEA Recording and Stimulation Protocols

Before processing, all extracellular data were digitally band-pass filtered (500–3000 Hz). In the stimulation experiments, biphasic positive-first voltage pulses were used with 200 µs phase width. The voltage for extracellular stimulation ranged between ±50 mV and ±300 mV.

#### Spontaneous Spike Amplitude Map

During the cultivation period, extracellular activity was monitored and recorded inside the incubator, while spontaneous firing activity was typically observed after one week in culture. One day prior to a patch clamp experiment, the complete array was scanned for spontaneous activity. Signals were recorded sequentially from 146 electrode configurations for 30–60 s per configuration, where each configuration consisted of a high-density electrode block (6 × 17 electrodes). Spikes were detected at every electrode by thresholding (threshold level of 5.5x signal standard deviations), and the negative-peak amplitudes for all electrodes were displayed in “spontaneous spike amplitude maps” (SSAMs, shown in Fig. 2a, Fig. 3a, and Fig. 7a) by using color coding. The SSAMs visualized the viability of the cultures as well as the position of spontaneously active neurons on the array.

#### Electrodes at local peaks in the SSAM

Electrodes with large spike amplitudes indicated that active neurons were located nearby, and, therefore, indicated suitable sites for recording and stimulation. Since some experiments required to record from or to stimulate many neurons, the respective recording and stimulation sites needed to be identified automatically based on the SSAM. We therefore used sites representing local peaks in the SSAM. First, all electrodes with negative peak-amplitudes exceeding a predefined threshold were selected. In a second step, if multiple neighboring electrodes had exceeded the threshold, only the electrodes closest to the local peak amplitude were further considered. The threshold value was set manually, while a MATLAB function provided the information of how many selected electrodes resulted for a particular value. Depending on the specific experiment, the threshold value was varied to yield the desired number of electrodes.

Isolated cells on the array typically yielded a single local peak coinciding with the location of the electrode yielding the largest signal amplitude. For aggregations of neurons, in contrast, the footprints of the cells largely overlapped. Therefore, the resulting number of local peaks in regions of neuron aggregation was significantly smaller than the number of cells. Avoiding the selection of many electrodes underneath aggregations of multiple neurons was particularly important for the stimulation experiments, as the probability was high that (a) an electrode would stimulate multiple cells simultaneously and (b) multiple electrodes would stimulate the same cell.

#### Spike Sorting

In order to identify which presynaptic neuron triggered individual PSPs at the patched cell, the extracellular recordings of spontaneous spiking activity were spike-sorted. We used the manually supervised spike sorting software Ultramegasort^54^. Ultramegasort can either process individual recording channels or consider multiple channels for the sorting. In the case of analyzing multiple channels, spikes are detected on every channel, and waveforms are then cut around the detection event on all the channels and combined by concatenation before feature extraction. We combined the signals from neighboring electrodes for the sorting, however, signals from distant electrode groups were sorted individually.

#### Randomized HD-MEA stimulation and identification of stimulation-triggered PSPs

Using the intracellular recording and the extracellular stimulation capability of the array, we investigated, which electrodes would stimulate neurons, which in turn elicited a PSP in the patched neuron. To this end, electrodes at local peaks in the SSAM were identified (threshold value set to yield approximately 50 local peaks). The electrodes were then randomly stimulated with biphasic stimuli of variable voltages.

Every electrode was stimulated with a series of stimulation voltages (typically 5 to 10 times per voltage), and the stimulations were applied at 4 Hz in a fully randomized order, while stimulation electrodes were always changed for two consecutive stimulations. After completion of the stimulation protocol, the intracellular traces of the patched cell 5 ms before and 50 ms after each individual stimulation pulse on every electrode were sequentially analyzed. Electrodes and the respective lowest stimulation voltages, which reliably evoked PSPs (for >80% of the applied stimuli) in the intracellular recordings, were then manually selected through a custom graphical user interface.

### Short- and Long-term Plasticity Measurements

Stimulation-triggered PSPs were identified with the randomized HD-MEA stimulation protocol described above. Short-term synaptic plasticity was measured by eliciting pairs of presynaptic APs on individual electrodes, separated in time by 50 ms. Different presynaptic neurons were sequentially stimulated at intervals of 600 ms. For each pair of EPSPs, the paired-pulse ratio (PPR) was calculated as $\frac{EPS{P}_{1}}{EPS{P}_{2}}$, where $EPS{P}_{1}$ and $EPS{P}_{2}$ denote the peak amplitude of the first and second PSP, respectively. The intracellular traces during paired pulses were averaged, and the timings of the first and second PSP peaks were identified. These timings were used to measure the amplitudes of the first and second PSPs for each individual trace. To calculate the PPR, only trials were considered for which the postsynaptic neuron was at rest (membrane potential (MP) < 60 mV) and did not depolarize significantly until the peak of the second PSP.

Long-term plasticity was induced by applying the intracellular tetanization (IT) protocol described in ref. 11 and visualized in Fig. 8b. It consisted of three trains (one per minute) of 10 bursts (1 Hz), where each burst was composed of five intracellular current pulses (5 ms) applied at 100 Hz. The amplitude was adjusted in order to elicit one AP per pulse. In order to measure plastic changes of the synaptic signals upon IT, EPSPs were measured by eliciting APs sequentially at the presynaptic neurons (1 s intervals). These measurements were carried out during 5 min before and during 20 min after the IT protocol.

## Electronic supplementary material

Supplementary Figure 1
