# Supplementary material for: Combination of High-density Microelectrode Array and Patch Clamp Recordings to Enable Studies of Multisynaptic Integration
Source: Sci Rep. 2017 Apr 20;7:978. doi: 10.1038/s41598-017-00981-4 (PMC5430511; doi:10.1038/s41598-017-00981-4)
Supplement: Supplementary file 1 — Supplementary Figure 1 [file 41598_2017_981_MOESM1_ESM.pdf]

Combination of High-density Microelectrode Array and Patch Clamp Recordings to Enable Studies of Multisynaptic Integration

Authors

David Jäckel, Douglas J. Bakkum, Thomas L. Russell, Jan Müller, Milos Radivojevic, Urs Frey, Felix Franke, and Andreas Hierlemann*

ETH Zurich, Department of Biosystems Science and Engineering, 4058 Basel, Switzerland

** Correspondence to: andreas.hierlemann@bsse.ethz.ch*

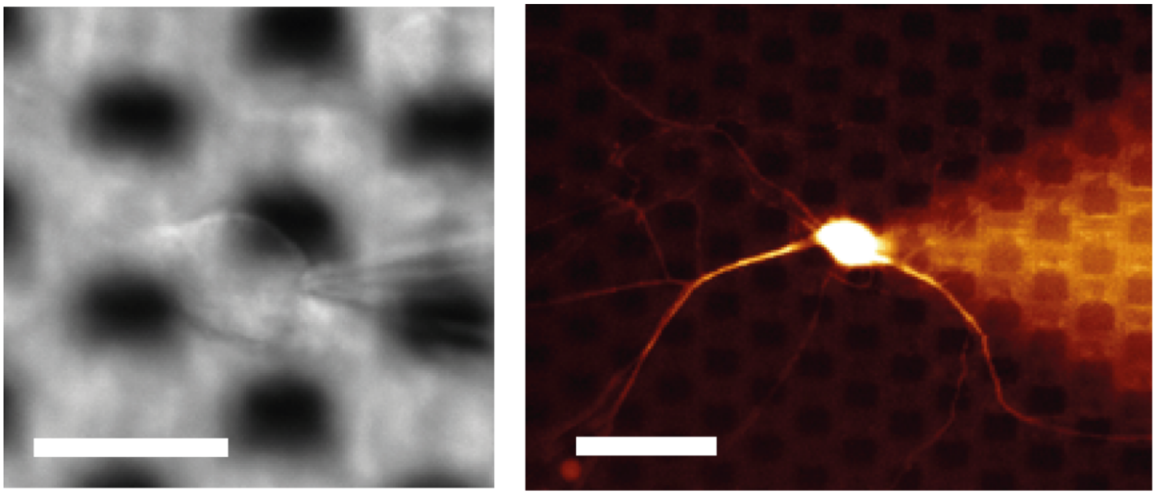

Supplementary Figure 1

Left: Bright field image of a patched neuron. In the background, the HD-MEA electrodes can be seen as black squares, scale bar: 20 µm. *Right:* Fluorescent image of the same neuron, scale bar: 50 µm.
